# Cbl-associated protein is tyrosine phosphorylated by c-Abl and c-Src kinases

**DOI:** 10.1186/1471-2121-10-80

**Published:** 2009-11-05

**Authors:** Inga Fernow, Ana Tomasovic, Ann Siehoff-Icking, Ritva Tikkanen

**Affiliations:** 1 Institute of Biochemistry, University of Giessen, Friedrichstrasse 24, 35392 Giessen, Germany; 2 Institute of Biochemistry II, University Clinic of Frankfurt, Theodor-Stern-Kai 7, 60590 Frankfurt am Main, Germany

## Abstract

**Background:**

The c-Cbl-associated protein (CAP), also known as ponsin, localizes to focal adhesions and stress fibers and is involved in signaling events. Phosphorylation has been described for the other two members of the sorbin homology family, vinexin and ArgBP2, but no data exist about the putative phosphorylation of CAP. According to previous findings, CAP binds to tyrosine kinase c-Abl. However, it is not known if CAP is a substrate of c-Abl or other tyrosine kinases or if phosphorylation regulates its localization.

**Results:**

We here show that CAP is Tyr phosphorylated by and interacts with both c-Abl and c-Src. One major phosphorylation site, Tyr360, and two minor contributors Tyr326 and Tyr632 were identified as Abl phosphorylation sites, whereas Src preferentially phosphorylates Tyr326 and Tyr360. Phosphorylation of CAP was not necessary for its localization to focal adhesions and stress fibers, but Tyr326Phe substitution alters the function of CAP during cell spreading.

**Conclusion:**

This is the first demonstration of phosphorylation of CAP by any kinase. Our findings suggest that coordinated action of Src and Abl might regulate the function of CAP and reveal a functional role especially for the Src-mediated Tyr phosphorylation of CAP in cell spreading.

## Background

CAP (c-Cbl-associated protein), also known as ponsin [1], is an adaptor protein which has been associated with regulation of the actin cytoskeleton, signaling through receptor tyrosine kinases and cell adhesion [2]. Numerous isoforms of CAP with different tissue distributions have been described in the literature, although their specific functions remain to be clarified [1,3-6]. Consistent with the function of CAP as an adaptor protein, most of these isoforms show a predominant localization with actin cytoskeleton, stress fibers, focal adhesions or cell-cell adhesion structures [7,8], but an isoform containing a nuclear localization signal has also been described [3].

CAP belongs to the so-called Sorbin homology (SoHo) protein family [2] together with vinexin-α and Arg-binding protein 2 (ArgBP2). All three members of this family contain in their N-terminal part one SoHo domain which was named after its homology to the gut peptide sorbin that participates in the regulation of absorption of water and electrolytes in the gall bladder [9]. However, the exact molecular function of the SoHo domain has only recently been elucidated. The SoHo domain of CAP was shown to be necessary for the formation of molecular complexes with the membrane raft-associated protein flotillin-1/reggie-2 (Flot-1) during insulin receptor signaling [10]. Upon insulin stimulation, CAP and c-Cbl are recruited by APS (Adapter Protein with pleckstrin homology and Src homology 2 domains) to insulin receptor [11], and c-Cbl becomes Tyr-phosphorylated. The CAP-c-Cbl complexes are then recruited to rafts by means of an interaction of the SoHo domain of CAP with Flot-1, which results in specific signaling events within raft membranes [10]. Later findings have suggested that a similar complex containing CAP/c-Cbl/Flot-1 would also play a role during signaling mediated by neurotrophic factor receptor TrkA [12]. In addition, other SoHo proteins have also been suggested to form SoHo-mediated signaling complexes with Flot-1 [13,14].

In addition to the SoHo domain, a characteristic feature of all these family members is the presence of three SH3 domains (referred to as SH3-A, -B and -C) in the C-terminal part of the protein [2]. SH3 domains are well known protein-protein interaction motifs that bind to proline-rich sequences and facilitate the formation of molecular complexes with various ligands [15]. The SH3 domains B and C of CAP/Ponsin have been shown to mediate the interaction with afadin which in turn is a binding partner of the transmembrane protein nectin in cell-cell adhesion structures that precede the formation of cadherin-based adherens junctions in epithelial cells [1,16]. The third SH3 domain of CAP also binds to c-Cbl, and focal adhesion kinase (FAK) has been reported to bind to SH3-B [5,7]. All three SoHo proteins directly interact with the actin associated protein vinculin [1,17-19], the localization of which largely overlaps that of the SoHo proteins in cell-matrix adhesions and stress fibers. The interaction with vinculin is mediated by the SH3 domains A and B in CAP [1].

In agreement with their role as cytoskeletal adaptor proteins, CAP, vinexin and ArgBP2 have been shown to be strongly associated with actin cytoskeleton and focal adhesions. Tyrosine phosphorylation of vinexin-α and ArgBP2 by c-Abl tyrosine kinase has previously been reported [20,21]. In both cases, the SH3 domains, especially SH3-C, were found to be important for this interaction. CAP has also been reported to bind to and coimmunoprecipitate with c-Abl in insulin stimulated hepatocytic cell line Hep3B, and in vitro experiments indicated that SH3-C in CAP would be a binding determinant of c-Abl [4]. However, no data are available if CAP is also phosphorylated by c-Abl and how the putative phosphorylation regulates the function of CAP. In addition, the possibility that CAP might be phosphorylated by other Tyr kinases known to be important for the regulation of the actin cytoskeleton and cell adhesions, such as the Src family kinases, remains to be investigated.

Here we show that CAP is indeed Tyr phosphorylated by both c-Abl and c-Src, and CAP can be coimmunoprecipiated with active forms of the kinases. The SH3 domains of CAP participate in the interaction in vivo but appear not to be the only binding determinants of c-Abl in CAP. We have identified Tyr360 in CAP as the primary phosphorylation site of c-Abl. In addition, Tyr632 appears to contribute to the phosphorylation of CAP by Abl, whereas Src phosphorylates both Tyr326 and Tyr360. Interestingly, mutation of Tyr326 altered the function of CAP during cell spreading, whereas exchange of Tyr360 or Tyr632 did not influence the spreading. However, none of the mutations affected the localization of CAP in focal adhesions or stress fibers.

## Results

### CAP is Tyr phosphorylated in vanadate treated Hep3B cells

**Figure 1:**
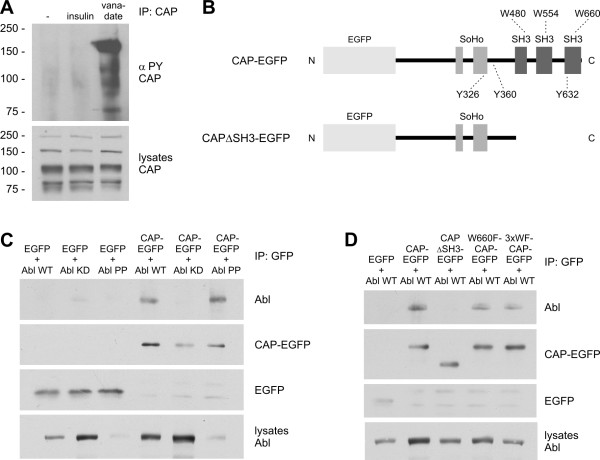
**CAP is phosphorylated by and coimmunoprecipitates with c-Abl**. A: Hep3B cells were starved, treated with insulin or vanadate, and CAP was immunoprecipitated with specific antibodies. Tyr phosphorylation of CAP was analyzed by Western blot. Endogenous CAP was phosphorylated after vanadate treatment but not in starved cells or after insulin stimulation. B: Schematic presentation of the CAP constructs based on the isoform CAP1 used in this study. See text for details. C: HeLa cells were cotransfected with CAP-EGFP or EGFP and either c-Abl WT, kinase-dead mutant (Abl KD) or constitutively active Abl (Abl PP). CAP was immunoprecipitated with GFP antibodies and coprecipitation of Abl was detected by means of Western blot (uppermost panel). Abl WT and PP were coprecipitated with CAP whereas Abl KD was not. D: Coprecipitation of Abl WT with various mutants of CAP. Abl WT was found to coprecipitate with full-length CAP, and somewhat less with the third SH3 domain mutant W660F and the 3 × WF mutant in which all three SH3 domains of CAP have been mutated. No coprecipitation was observed with CAPΔSH3 or EGFP only.

A previous report showed that CAP coimmunoprecipitates with c-Abl from insulin stimulated Hep3B cells [4]. To study if CAP is also phosphorylated in these cells, we immunoprecipitated endogenous CAP from cells stimulated with insulin or treated with vanadate. A signal for Tyr phosphorylated CAP was detected in vanadate-treated cells, whereas nonstimulated or insulin-treated cells showed no signal (Figure 1A). Several isoforms of CAP were expressed in Hep3B cells, but mainly the 150 kDa isoform was found to be Tyr phosphorylated.

### CAP is phosphorylated by and interacts with c-Abl

Since Hep3B cells are extremely difficult to transfect and endogenously contain several isoforms of CAP, we chose HeLa cells for further experiments since they do not express any endogenous CAP [8]. For this, we used overexpression of N-terminally GFP-tagged isoform CAP1 (GenBank: U58883) which contains a SoHo domain and three SH3 domains C-terminally to it (Figure 1B). Coimmunoprecipitation of various activity mutants of c-Abl (WT: wild-type, Abl KD: kinase-dead, Abl PP: constitutively active) with CAP-EGFP was analyzed. Abl WT and PP were found to coimmunoprecipitate with CAP, whereas the Abl KD was not (Figure 1C). Although the expression of Abl PP was low in cell lysates (lowermost panel) due to toxicity of high level expression, a similar amount to Abl WT was detected in immunoprecipitates, indicating that a higher percentage of the cellular Abl PP was associated with CAP.

It has been reported that the third SH3 domain (SH3-C) of CAP binds Abl in vitro. We thus used CAP mutants carrying mutations or deletions in the SH3 domain region (Figure 1B) to study the coimmunoprecipitation of WT Abl. Although the full-length CAP coprecipitated Abl, the C-terminal deletion mutant of CAP, ΔSH3, missing all three SH3 domains, did not precipitate any Abl (Figure 1D). However, point mutants W660F, carrying a WF substitution in the absolutely conserved, critical Trp residue in SH3-C, and the mutant 3 × WF, in which all three SH3 domains of CAP have been mutated, both were capable of coprecipitating reduced but still considerable amounts of Abl.

**Figure 2:**
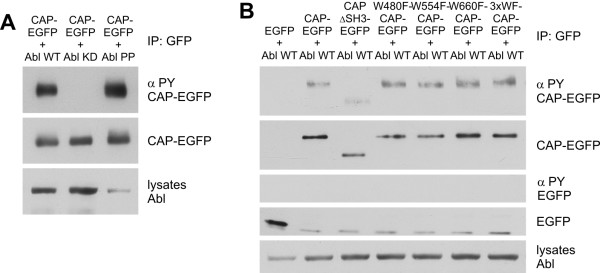
**Kinase activity of Abl is necessary for the phosphorylation of CAP**. HeLa cells were cotransfected with WT and mutants of CAP-EGFP and Abl. CAP was immunoprecipitated with GFP antibodies and the phosphorylation status was detected with anti-phospho-Tyr antibodies in Western blots. A: Full-length CAP was phosphorylated by Abl WT and Abl PP but not by Abl KD. B: WT CAP, all single WF and the 3xWF SH3 domain mutants were phosphorylated upon coexpression of c-Abl, whereas the CAPΔSH3 mutant was not.

We next studied if CAP would be tyrosine phosphorylated by c-Abl. Upon coexpression of Abl WT and PP, a high degree of Tyr phosphorylation of CAP was detected, whereas no phosphorylation was deteted in cells cotransfected with Abl KD (Figure 2A). The phosphorylation of CAP upon coexpression of Abl PP was higher than with Abl WT, despite the fact that the expression level of Abl PP was much lower than that of Abl WT.

Having established that CAP is a substrate of Abl kinase, we studied the phosphorylation of CAP mutants containing substitutions in the SH3 domain region (Figure 2B). Consistent with its inability to bind to Abl, CAP ΔSH3 was phosphorylated very little upon Abl coexpression. However, the WF point mutants containing substitutions either in one of the SH3 domains (W480F: SH3-A, W554F: SH3-B, W660F: SH3-C) or in all three SH3 domains (3xWF) showed a phosphorylation comparable to or even higher than WT CAP, again consistent with their ability to associate with Abl. These results would suggest that although functional SH3 domains are not necessary for the tyrosine phosphorylation of CAP by c-Abl, the C-terminal part of CAP is required for a productive interaction.

**Figure 3:**
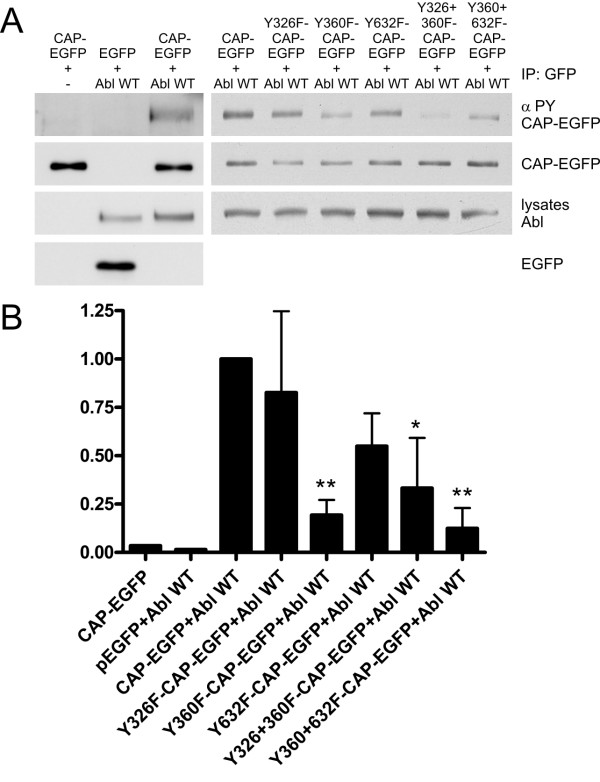
**Y360 in CAP is the major phosphorylation site of c-Abl**. A: Tyr residues 326, 360 and 632 in CAP were mutated into Phe and their phosphorylation upon c-Abl coexpression was analyzed as in Figure 2. No phosphorylation of CAP was detected in the absence of coexpressed Abl kinase. Y360F, Y632F, Y326F+Y360F and Y360+Y632F exhibited a reduced Tyr phosphorylation whereas Y326F was comparable to WT CAP. B: Quantitative analysis of the phosphorylation summarizing data from three experiments. The phosphorylation signals were normalized with the expression level of the respective variants and given as % of WT CAP phosphorylation. Error bars show standard deviation.

We then sought to identify the Tyr residues in CAP that would be phosphorylated by Abl. It has been shown that Abl preferably phosphorylates substrates with the consensus sequence I/L/V-**Y**-(X)_1-5_-P/F around the Tyr residue to be phosphorylated [22]. Such a sequence was found around three Tyr in CAP, namely Y326, Y360 and Y632, which were mutated into Phe residues. Phosphorylation of CAP upon coexpression of Abl was again analyzed (Figure 3). No phosphorylation signal was detected in cells transfected with CAP alone or with Abl and EGFP. The mutant Y326F was found to be phosphorylated at about equal level as the WT CAP, and Y632F showed a moderate reduction of phosphorylation (55% ± 17% of WT level). However, Y360F mutant showed a clearly reduced phosphorylation (19% ± 8.0%). Interestingly, the double mutants Y326F+Y360F (33% ± 26%) and Y360F+Y632F (12% ± 11%) both exhibited a low degree of phosphorylation. These data suggest that Y360 is the main phosphorylation site, but Y632 also seems to contribute to the phosphorylation of CAP by Abl.

### Mutation of Tyr 326, 360 or 632 does not affect the localization of CAP into focal adhesions and stress fibers

**Figure 4:**
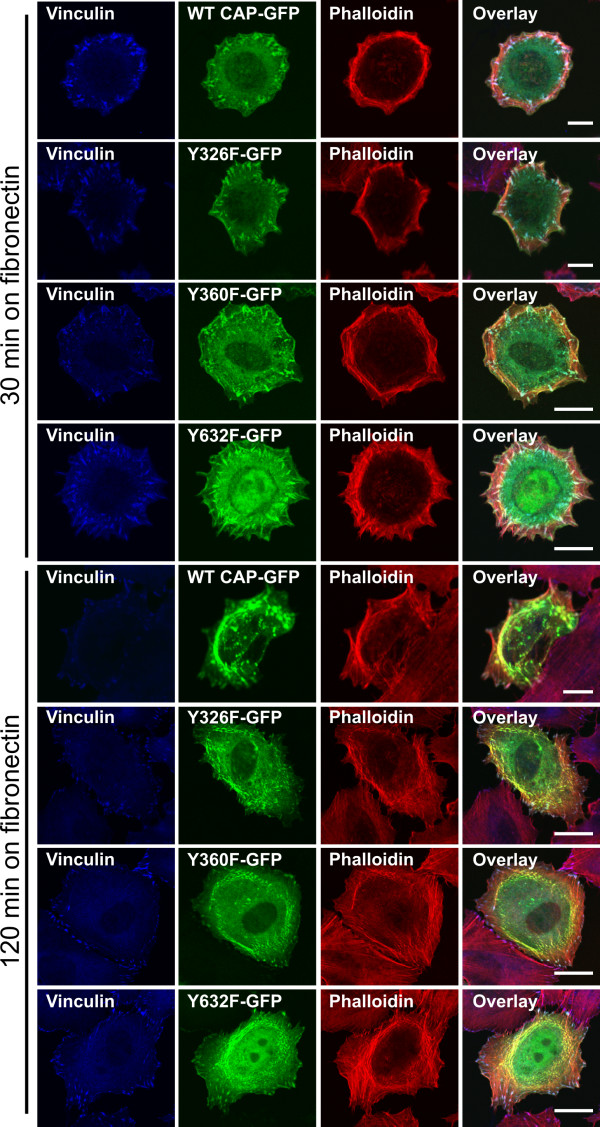
**Tyrosine mutations do not alter the localization of CAP into focal adhesions and stress fibers**. Cells transfected with CAP-EGFP, Y326F, Y360F or Y632F mutant were seeded out on fibronectin coated coverslips, allowed to adhere for 30 min or 120 min, fixed and stained for vinculin (blue) and phalloidin (red). WT CAP, Y326F and Y360F mutant were found to show a similar overall localization, whereas Y632F mutant exhibited some nuclear staining, in addition to focal adhesions and stress fibers. All CAP variants showed two localization patterns, either colocalizing with vinculin in cell matrix adhesions after shorter spreading times (30 min) or with actin fibers after longer times of spreading (120 min).

CAP has been reported to localize in stress fibers and to colocalize with vinculin in focal adhesions [1]. When WT CAP transfected cells were seeded out on fibronectin and allowed to spread (Figure 4, upper panel), after 30 min we observed a localization of CAP (green) in focal adhesions at the cell periphery where it colocalized with vinculin (blue) but only poorly with cortical actin fibers (red). After the cells had spread out for 120 min (Figure 4, lower panel), a minor fraction of CAP colocalized with vinculin (blue) in focal adhesions, whereas most of the CAP protein was detected in stress fibers, colocalizing with actin (red). We could not detect any clear difference in the localization of the Y326F, Y360F (Figure 4) or Y326F+Y360F mutant (not shown) as compared to the WT CAP. However, mutant Y632F was localized both in focal adhesions and stress fibers (Figure 4) but additionally showed some nuclear localization. These results imply that Abl mediated phosphorylation is not necessary for association of CAP with focal adhesions or stress fibers. However, during the spreading of the cells, the localization of CAP seems to dynamically change from focal adhesions to stress fibers, according to the spreading state of the cells.

### Y326F mutant exhibits an inhibitory effect on cell spreading

**Figure 5:**
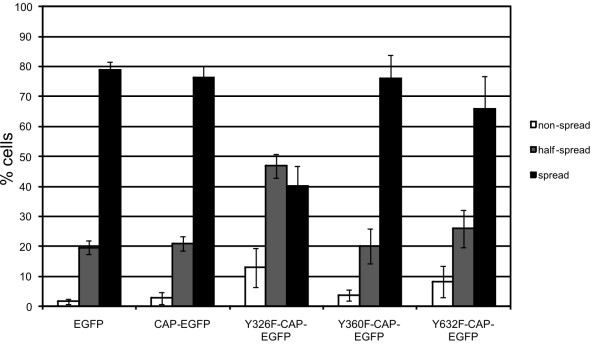
**Y326F substitution in CAP impairs cell spreading on fibronectin**. HeLa cells transfected with various CAP-EGFP constructs were allowed to adhere to fibronectin-coated coverslips for 25 min, then washed and fixed. The cells were scored on the basis of their spreading status as non-spread, half-spread or fully spread. WT CAP, Y360F or Y632F mutant showed no effect on cell spreading as compared to EGFP transfected cells, whereas Y326F mutant exhibited a slower spreading rate. The graph shows the results of 3 independent experiments with at least 100 cells scored in each. Error bars = SD.

Although the localizations of CAP and the Tyr mutants were similar during various time points of cell spreading (Figure 4), these data provide only limited information on the role of phosphorylation during active, dynamic cell spreading. In order to study the possible functional role of the Tyr phosphorylation, we performed spreading assays of transfected HeLa cells on a fibronectin matrix (Figure 5). In this assay, the cells were allowed to spread on fibronectin coated surface for 25 min, fixed and scored for their spreading state in three categories (non-spread for round cells that were barely attached; half-spread for cells that showed filopodia and had begun to flatten; spread for flat cells with well-spread lamellipodia). The cells transfected with EGFP, WT CAP, Y360F and Y632F mutant all showed a high degree (70-80%) of fully spread cells. However, in the case of the Y326F mutant, a clearly inhibitory effect on the spreading of the cells was detected, with only 40% of the cells exhibiting a fully spread phenotype and some 45% showing a half-spread morphology with many filopodia-like protrusions. Thus, mutation of Tyr326 in CAP appears to have an inhibitory effect on cell spreading.

### CAP is phosphorylated by c-Src

**Figure 6:**
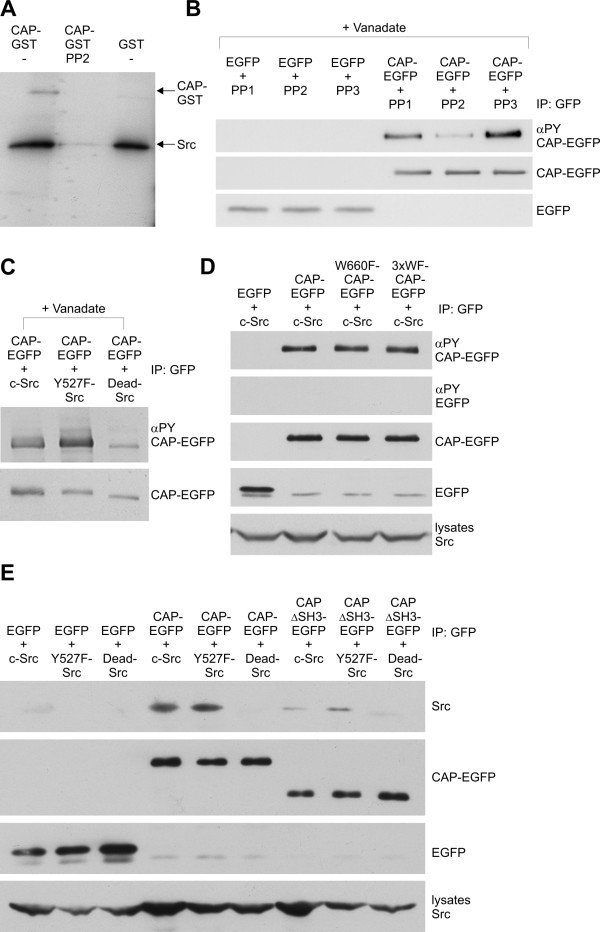
**CAP is a substrate of Src kinase**. A: Purified CAP-GST or GST was incubated with recombinant, purified Src kinase and [γ-^32^P]-ATP, run into SDS-PAGE gel and analyzed by autoradiography. Specific phosphorylation of CAP-GST could be detected after incubation with active Src but not after inhibition of Src activity with PP2. Autophosphorylation of Src was used as an internal control for successful inhibition. B: CAP-EGFP or EGFP were immunoprecipitated from cells treated with vanadate and either Src inhibitors PP1 or PP2 or a non-inhibiting analogue PP3. Inhibition of endogenous Src activity clearly reduced the Tyr phosphorylation of CAP-EGFP. C: Coexpression of active forms of Src kinase (c-Src or Y527F-Src) notably increased the Tyr-phosphorylation of CAP-EGFP as compared to cells coexpressing the inactive Src mutant (Dead-Src). D: Intact SH3 domains of CAP are not necessary for the phosphorylation of CAP by Src. CAP-EGFP or CAP mutants carrying mutations in the SH3 domains were coexpressed and the phosphorylation of CAP was studied after immunoprecipitation. Mutation of the critical tryptophane in the third SH3 domain (W660F-CAP-EGFP) or in all three SH3 domains (3xWF-CAP-EGFP) exhibited no effect on the degree of Tyr phosphorylation of CAP by Src. E: Active forms of Src (c-Src and Y527F-Src) were coprecipitated with WT CAP and to a severely reduced degree with the ΔSH3 mutant of CAP, whereas kinase dead Src did not associate with either the WT or ΔSH3 CAP.

Since Tyr326 was not a major c-Abl phosphorylation site, we sought to identify a putative other kinase that might be involved in the phosphorylation of Y326 in CAP. Src kinases are good candidates for this since they have been shown to play an important role not only in signal transduction by growth factors but also in the regulation of cell matrix adhesion. Recombinant Src kinase was indeed capable of phosphorylating the purified CAP-GST protein in an *in vitro *phosphorylation assay, whereas inhibition of Src activity by means of PP2 completely abrogated CAP phosphorylation in the *in vitro *assay (Fig 6A) and severely impaired it in transfected cells (Figure 6B). When cells were transfected with CAP-EGFP and Src kinase constructs (c-Src: WT Src, Y527F: constitutively active Src, Dead-Src: kinase-inactive Src), an increased phosphorylation of CAP variants was detected, correlating with the activity level of Src (Figure 6C). The small amount of phosphorylated CAP detected in cells transfected with Dead-Src is most likely due to phosphorylation by endogenous c-Src and enhancement upon vanadate incubation. As with c-Abl, phosphorylation of CAP by c-Src appears not to depend on functional SH3 domains since the SH3 domain mutants of CAP were found to be Tyr phosphorylated to a comparable level as the WT CAP (Figure 6D and not shown for W480F and W554F). As with the Abl kinase, only active forms of Src were found to coprecipitate with CAP, and deletion of the SH3 region of CAP resulted in severely reduced coprecipitation of Src with CAP (Figure 6E).

**Figure 7:**
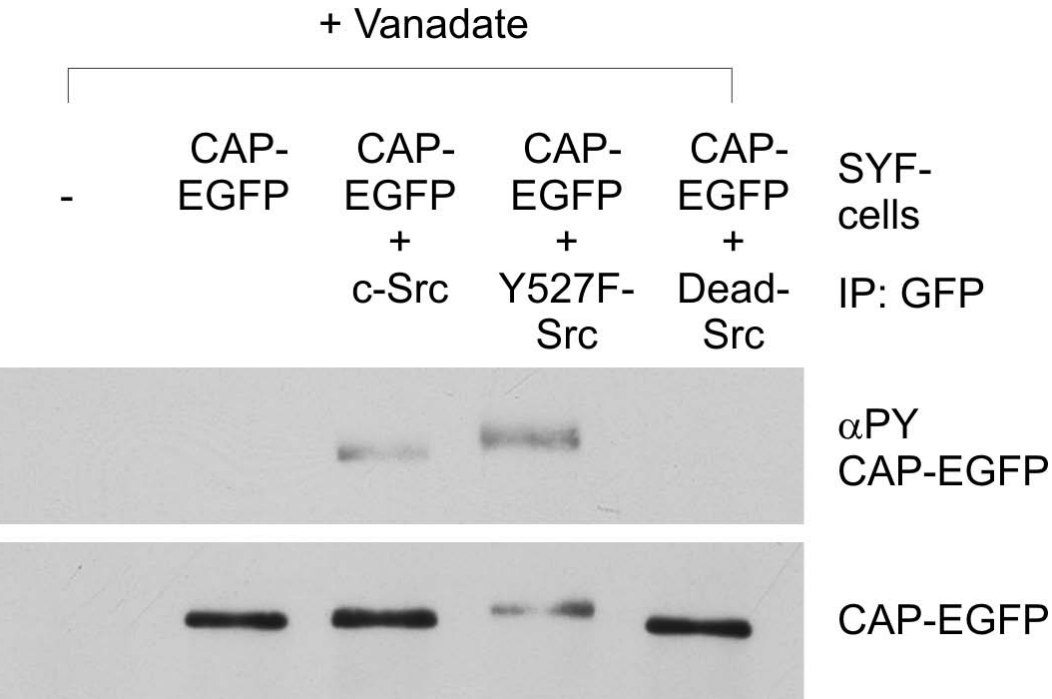
**Phosphorylation of CAP in SYF cells can be rescued upon expression of active forms of Src**. Embryonic fibroblasts from the triple knock-out mouse for Src, Yes and Fyn (SYF cells) were transfected with CAP-EGFP together with various forms of Src kinase and stimulated with vanadate. No Tyr phosphorylation of CAP could be detected in cells expressing only CAP-EGFP or the inactive Src (Dead-Src). Phosphorylation of CAP was rescued upon coexpression of either c-Src or the constitutively active mutant Y527F-Src.

To corroborate these findings, mouse embryonal fibroblasts genetically ablated for the ubiquitous Src kinases Src, Yes and Fyn (SYF cells) [23,24] were transfected with CAP-EGFP. In these cells, no phosphorylation of CAP-EGFP could be detected. However, phosphorylation of CAP could be rescued upon coexpression of WT and constitutively active Src, but not of the kinase dead Src. (Figure 7).

**Figure 8:**
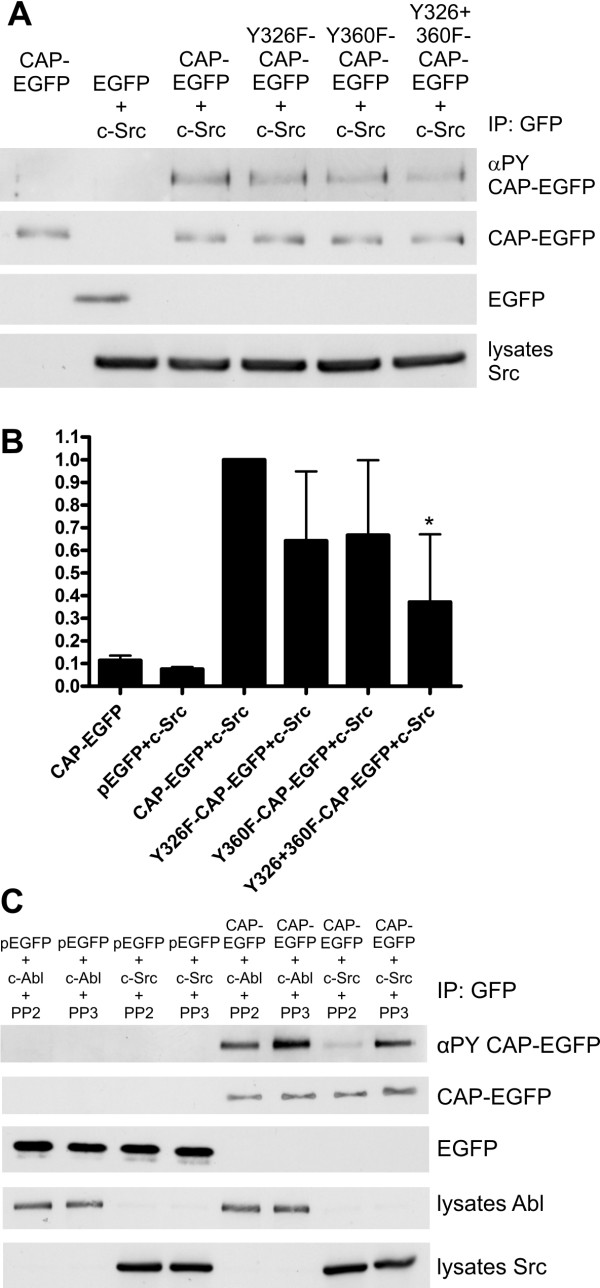
**Tyr326 and Tyr360 are the major phosphorylation sites of Src**. A: CAP-EGFP and its mutant variants were transfected into HeLa cells together with c-Src and the phosphorylation of CAP was analyzed after immunoprecipitation. Tyr phosphorylation of the Y326F and Y360F mutant was reduced as compared to the WT CAP. An even lower level of phosphorylation could be detected for the Y326F+Y360F mutant. B: Quantification of the phosphorylation of CAP upon c-Src copexpression. Shown is the summary of the results of four independent experiments, error bars show standard deviation. C: c-Abl or c-Src was transfected into HeLa cells together with CAP-EGFP or EGFP, and the cells were treated with the Src inhibitor PP2, which does not inhibit Abl kinase at this concentration, or with non-inhibiting analogue PP3. In c-Abl transfected cells, inhibition of Src activity results in reduced phosphorylation of CAP, whereas in c-Src transfected cells, no phosphorylation could be detected after PP2 treatment.

In order to identify the residues phosphorylated by Src, CAP-EGFP and its Tyr-mutated forms were coexpressed with c-Src in HeLa cells (Figure 8A). Substitution of Y326 or Y360, which represents the main phosphorylation site of c-Abl, alone only moderately impaired the phosphorylation of CAP by Src. However, the double mutant Y326+360F showed a clearly reduced level of Tyr phosphorylation, indicating that tyrosines 326 and 360 both might be phosphorylation sites of Src in CAP. Substitution Y632F showed no effect on the phosphorylation of CAP by Src (data not shown).

Since Y360 is also the major phosphorylation site of Abl in CAP, c-Src and c-Abl kinases could cooperate for the phosphorylation of CAP. To test this, CAP-EGFP was cotransfected with either c-Abl or c-Src, and the cells were treated with the Src inhibitor PP2 or a non-inhibitory control compound PP3. PP2 was used since, in contrast to PP1 and many other Src inhibitors, it does not inhibit Abl kinase activity in the concentration used here. Inhibition of Src activity in c-Abl transfected cells resulted in a reduction of CAP phosphorylation, whereas in Src transfected cells, no phosphorylation of CAP could be detected upon use of PP2. These results would imply that there is some cooperativity of CAP phosphorylation between c-Src and c-Abl.

## Discussion

We have here shown that endogenously expressed CAP can be Tyr phosphorylated in Hep3B hepatocytes, whereas previous findings from others have demonstrated that in these cells, CAP can be coimmunoprecipitated with c-Abl kinase [4]. Although other members of the SoHo protein family have been shown to be phosphorylated by c-Abl [20,21], our report is the first demonstration of Tyr phosphorylation of CAP by any kinase. CAP has been shown to play a role in membrane receptor signal transduction, e.g. insulin and neurotrophic factor signaling [10,12]. We were not able to detect phosphorylated CAP in insulin treated Hep3B cells, whereas vanadate stimulation resulted in robust Tyr phosphorylation of CAP. This might indicate that the phosphorylation of CAP during signaling is regulated by rapid dephosphorylation by phosphatases, and thus phosphorylation can only be detected upon inhibition of phosphatase activity by vanadate. It will be of interest to determine if CAP is phosphorylated in other cell types during signaling processes, and if so, which kinases and phosphatases mediate the phosphorylation and dephosphorylation.

The third SH3 domain of CAP (SH3-C) has previously been shown to bind to c-Abl in vitro [4]. According to our results, the delta-SH3 mutant of CAP missing all three SH3 domains was neither capable of coprecipitating c-Abl nor phosphorylated by it. However, we observed only a slightly reduced coprecipitation of c-Abl with the mutants in which either SH3-C or all three SH3 domains of CAP were mutated. As the Trp substitutions render SH3 domains incapable of binding their poly-Pro containing recognition signals, these results would suggest that although the binding site of Abl in CAP is located in the C-terminal part containing the SH3 domains, there have to be determinants other than the SH3 domains, e.g. the linker regions in between, which are important for the interaction of Abl with CAP. Interestingly, the linker region between SH3-B and -C contains a putative Pro-rich sequence (PQQP) which might function as a binding site for the SH3 domain of c-Abl, thus facilitating a dual interaction mediated by SH3 domains/Pro-rich regions in both proteins. A similar binding mode has also been implicated between vinexin and c-Abl [20]. However, mutation of the PQQP motif into AEEA did not significantly reduce the phosphorylation of CAP in the presence of intact SH3 domains (data not shown). Although this would rather speak against the role of the PQQP motif in the binding of Abl, an interaction mediated by more than one determinant would also be supported by the fact that the constitutively active Abl-PP mutant coprecipitated with CAP more than c-Abl. In Abl-PP, two Pro residues in the linker region between the SH2 and kinase domain have been mutated so that the protein acquires an open conformation [25] and might be able to bind more efficiently to its substrate, CAP. On the other hand, since phosphorylation of CAP was readily observed in cells coexpressing WT Abl and CAP, this might imply that binding of CAP to c-Abl could even increase the activity of the kinase which is under tight regulation in normal cells (see [26] for review). However, further experiments will be required to clarify the exact binding mechanism between CAP and Abl.

We have here identified Tyr360 in CAP as a major phosphorylation site by c-Abl, although Tyr 632 also might contribute since its substitution in combination with the Y360F mutation reduced the phosphorylation of CAP to a very low level. Both Tyr conform to the consensus c-Abl phosphorylation motif. Interestingly, in mouse vinexin, the major phosphorylation site of c-Abl is provided by Y127 with some possible contribution by Y269 and Y394 [20]. However, different from Y360 in CAP, Tyr127 of vinexin does not seem to reside in a consensus phosphorylation motif for c-Abl.

CAP localizes to focal adhesions, where it can bind vinculin, FAK, paxillin and filamin C and to actin stress fibers, which is probably, at least to some degree, mediated by different sets of binding partners [1,7,8,27,28]. In fibroblasts, overexpression of CAP has been described to slow down the spreading of cells onto extracellular matrix [8]. However, according to our data, CAP does neither inhibit nor enhance the spreading of epithelial cells onto fibronectin. This could be explained by the differences in the modes of regulation of focal adhesions and cell spreading between different cell types or by the presence of binding partners of CAP in fibroblasts that result in inhibitory effects. Although c-Abl is an important regulator of cytoskeletal remodeling and many of its substrates are known to participate in the coordination of actin-dependent processes, we could not observe any effect on cell spreading when Y360, the major c-Abl phoshorylation site of CAP, or Y632 were mutated to Phe. Both mutants were also localized in both focal adhesions and stress fibers in a very similar way to the WT CAP protein. The Y632F mutant also exhibited some nuclear localization which is at present difficult to explain. Although a nuclear form of CAP, termed R85Fl [3], has been described, the isoform used here does not contain the nuclear localization signal shown to mediate the nuclear transport of R85Fl. Thus, the nuclear translocation of Y632F mutant might be mediated by its interaction with another protein that is capable of being transported into the nucleus, as has been shown the case with teneurin-1 and CAP [29].

Since substitution of the major Abl phosphorylation sites in CAP resulted in normal spreading of the cells, phosphorylation of CAP by c-Abl does not seem to play a major role in this process. However, the mutation of Y326 slowed down the spreading, indicating that phosphorylation of this Tyr by another kinase might be important for the regulation of CAP function during cell spreading. Indeed, we could show that Y326 is phosphorylated by c-Src, another non-receptor Tyr kinase that regulates many actin dependent cellular processes, including focal adhesion assembly and spreading [30,31]. If phosphorylation of Y326 by c-Src indeed plays a role in the regulation of cell spreading by CAP still needs to be clarified in more detail in future studies.

As with Abl, the binding and phosphorylation of CAP by Src was severely compromized by deletion of the C-terminal SH3 region but not by mutations in the critical Trp residued in the SH3 domains of CAP. Thus, also in the case of Src, the linker regions between the SH3 domains and the Pro rich sequences might mediate the interaction between the kinase and its substrate. The main phosphorylation sites Y326 and Y360 do not reside in the SH3 region but more N-terminally to it, and thus the reduced phosphorylation of ΔSH3 CAP by Src and Abl is unlikely to be simply due to deletion of phosphorylatable Tyr residues. In addition, mutation of six tyrosines within the SH3 domain region of CAP (Y527, Y587, Y592, Y632, Y678 and Y638) which all fit well to the consensus phosphorylation sequence of Src did not affect the phosphorylation of CAP by c-Src (our unpublished data). Thus, the SH3 region is more likely to be important for the substrate-kinase interaction rather than providing substrate tyrosines.

Substitution of Y360, the major Abl phosphorylation site, alone only moderately impaired the phosphorylation of CAP by Src. However, the double mutant Y326F/Y360F showed a severely impaired phosphorylation by both Src and Abl, implicating that both kinases might actually cooperate to achieve the full degree of phosphorylation of CAP. It could be speculated that Src plays only an indirect role in the phosphorylation of CAP by facilitating Abl activation and thus increasing Abl-mediated CAP phosphorylation. However, our in vitro phosphorylation assay with purified proteins showed that CAP is a direct substrate of Src. Furthermore, CAP interacted with Src in coimmunoprecipitation experiments, and Y326 fits well to the consensus phosphorylation sequence of c-Src. Thus, it appears that both c-Src and c-Abl are capable of directly phosphorylating tyrosines 326 and 360 in CAP, but each show a preference to a different Tyr. However, inhibition of Src activity in cells expressing c-Abl resulted in slightly reduced phoshorylation of CAP. Thus, both kinases appear to contribute to the phosphorylation and thereby generate binding sites for SH2-containing proteins which would link CAP downstream towards processes resulting in actin remodeling. The details of the interplay between Src and Abl in the phosphorylation of CAP should be clarified in future studies in order to understand the role of phosphorylation in the regulation of the function of CAP.

## Conclusion

Taken together, our results show that CAP is a direct substrate of both c-Abl and Src kinase which each phosphorylate CAP at least in one Tyr residue. However, both kinases appear to have their preferential phosphorylation sites in CAP. The details of the functional regulation of CAP by Abl- and Src-mediated phosphorylation still need to be clarified in future studies. The SoHo proteins show a very similar localization pattern, share many binding partners and are all phosphorylated by c-Abl. So far, the details of the functional significance of Abl-mediated phosphorylation have not been characterized for any of the SoHo proteins. Thus, it will be important to address the specific downstream events that result from the phosphorylation of each of the SoHo family members and which thus mediate the functional differences between these proteins. Further work will also show how phosphorylation of CAP by Abl and Src contributes to its function.

## Methods

### Antibodies

Antibodies were as follows: immunoprecipitation: polyclonal anti-CAP (Biomol); polyclonal anti-GFP (Clontech); Western blot: monoclonal anti-GFP (Roche); monoclonal anti-c-Abl (Sigma); monoclonal anti-Src (Upstate), monoclonal anti-phospho-Tyr (P-Tyr-100, Cell Signaling); immunofluorescence: monoclonal anti-vinculin (Sigma).

### Plasmid cloning and mutagenesis

The isoform CAP1 (GeneBank U58883) containing a SoHo domain and three C-terminal SH3 domains was used in this study. CAP-EGFP was PCR amplified from CAP-Flag (kind gift of A. Saltiel, Michigan, USA) and cloned into the pEGFP-C1 vector (Clontech). CAP-EGFP was used as a template to create the CAPΔSH3-EGFP construct. Point mutations (W to F; Y to F) were generated with QuikChange Site-Directed Mutagenesis Kit (Stratagene). The c-Abl and c-Src constructs were a kind gift of I. Dikic (Frankfurt am Main, Germany).

### Cell culture and transfection

Hep3B, SYF (ATCC: CRL-2459) and HeLa cells were cultured in Dulbecco's modified Eagle's medium supplemented with 10% fetal calf serum. Transfections were performed with Metafectene (Biontex, Munich, Germany). Assays were performed 24-48 h post-transfection.

### Insulin stimulation, Src inhibitor treatment and immunoprecipitation

Hep3B cells were pretreated with 100 nM insulin for 5 min or with 100 μM sodium ortho-vanadate for 30 min. In some experiments (as marked in figures), transfected HeLa cells were pretreated with 100 μM ortho-vanadate for 25 min to induce phosphorylation. Src inhibition was accomplished by incubating the cells with 5 μM PP1, PP2 or PP3 (Calbiochem) for 25 min. For detection of phosphorylated proteins, cells were lyzed in lysis buffer (100 mM Tris pH 8.0, 150 mM NaCl, 1 mM MgCl_2_, 1% Triton X-100, 60 mM n-Octylglucoside), supplemented with Protease Inhibitor Cocktail (Sigma) and 1 mM vanadate. Lysates were incubated at 4°C for 16 h with Protein A Dynabeads (Invitrogen) precoupled with the antibody. Beads were washed with lysis buffer and PBS. The precipitated proteins were solubilized into 1× loading buffer by incubation at 90°C for 3 min. Proteins were separated by SDS-PAGE and subjected to immunoblotting with specific antibodies.

### Coimmunoprecipitation

Cells were lysed in coimmunoprecipitation buffer (50 mM Tris pH 7.6, 150 mM NaCl, 2 mM EDTA, 1% NP-40), supplemented with Protease Inhibitor Cocktail (Sigma). The lysates were precleared with 3× 50 μl Pansorbin beads (Calbiochem). After immunoprecipitation (see above), the Dynabeads were washed six times with TBST (10 mM Tris pH 8.0, 100 mM NaCl, 0.05% Tween 20) and once with 0.1× PBS. Otherwise the samples were processed as described above.

### *In vitro *phosphorylation assay

25 ng of recombinant, purified GST fusion protein was incubated in kinase buffer (10 mM Hepes pH 7.4, 5 mM MgCl_2_, 0,2 mM EDTA) with 50 ng recombinant, active human c-Src kinase (PHO3134, Biosource, Karlsruhe, Germany) and 1 μCi [γ-^32^P]-ATP (2 mM) in a total volume of 50 μl. After 15 min at 30°C, the reaction was stopped by adding 15 μl loading buffer, and the samples were denatured by incubating at 90°C for 3 min. Thereafter, the samples were run on SDS-PAGE, the gel was fixed in 25% isopropanol/10% acetic acid and dried. The samples were visualized by means of autoradiography on X-ray films.

### Immunofluorescence

Cells were fixed with 4% paraformaldehyde for 20 min, permeabilized with 50 μg/ml digitonin, blocked with 1% bovine serum albumin in PBS, followed by labeling with a primary antibody and a secondary Cy5-conjugated antibody. Actin cytoskeleton was stained with AlexaFluor594-Phalloidin (Molecular Probes). Cells were embedded in Gel/Mount (Biomeda) with 50 mg/ml 1,4-diazabicyclo(2,2,2)octane. Images were taken with a confocal laser-scanning microscope (Zeiss LSM 510 Meta), exported and processed with CorelDraw. Except for contrast and brightness adjustment for some images, no other electronic manipulations were undertaken. The images in Figure 4 represent single confocal slices of 1 μm thickness.

### Cell spreading assay

Cell spreading assays were carried out as described before [32], except that only 50.000 cells per well were seeded out on fibronectin coated glass chambers. After 25 min of spreading, the cells were washed, fixed and embedded as above. The spreading status of cells was scored using a fluorescence microscope. The cells were divided into three categories: non-spread (round but attached, no filopodia), half-spread (roundish, filopodia-like protrusions) and fully spread (flat, no filopodia-like protrusions, prominent lamellipodial structures). For images of cells representing each category, see [32].

### Quantification and statistical analysis

Unless otherwise stated, all experiments were performed at least three times. Quantification in Figs. 3 and 8 was done densitometrically. The signals for phosphorylation were corrected with the total amount of the respective CAP variant and expressed as % of the signal observed for the WT CAP which was defined as 100%. Results shown represent the mean of values plus standard deviation. Statistical analysis was done using one way ANOVA (Bonferroni test). * p < 0.05; ** p > 0.01

## Abbreviations

CAP: c-Cbl associated protein; Cbl: Casitas B-lineage lymphoma proto-oncogene; SoHo: Sorbin homology; ArgBP2: Arg-binding protein 2; Flot-1: Flotillin-1; TrkA: Tyrosine kinase receptor A; SH3: Src homology 3 domain; FAK: Focal adhesion kinase; EGFP: Enhanced Green Fluorescent Protein; PBS: Phosphate Buffered Saline; SDS-PAGE: sodium dodecyl sulfate polyacrylamide gel electrophoresis; CoIP: coimmunoprecipitation; NP-40: Nonidet P-40; TBST: Tris-Buffered Saline Tween-20.

## Authors' contributions

IF and AT performed the experiments, constructed the figures and participated in writing of the manuscript. AS-I generated some of the constructs used in this study and performed preliminary experiments. RT generated some of the constructs, performed the microscopy, constructed and coordinated the study and was responsible for writing the paper. All authors read and approved the final manuscript.
